# Vesicles: New Advances in the Treatment of Neurodegenerative Diseases

**DOI:** 10.3390/ijms252312672

**Published:** 2024-11-26

**Authors:** Domenico Nuzzo, Antonella Girgenti, Laura Palumbo, Flores Naselli, Martina Bavetta, Giovanni Marfia, Pasquale Picone

**Affiliations:** 1 Institute for Biomedical Research and Innovation, CNR, Via U. La Malfa 153, 90146 Palermo, Italy; antonella.girgenti@irib.cnr.it (A.G.); laura.palumbo@irib.cnr.it (L.P.); flores.naselli@irib.cnr.it (F.N.); martina.bavetta@irib.cnr.it (M.B.); 2 Department of Biological, Chemical and Pharmaceutical Sciences and Technologies (STEBICEF), University of Palermo, Viale delle Scienze Building 16, 90128 Palermo, Italy; 3 Laboratory of Experimental Neurosurgery and Cell Therapy, Neurosurgery Unit, Foundation IRCCS Ca’ Granda Ospedale Maggiore Policlinico, 20122 Milan, Italy; giovanni.marfia@policlinico.mi.it

**Keywords:** neurodegenerative diseases, nanotechnologies, extracellular vesicles, artificial vesicles

## Abstract

Neurodegenerative diseases are characterized by brain lesions that limit normal daily activities and represent a major challenge to healthcare systems worldwide, with a significant economic impact. Nanotechnology is the science of manipulating matter at the nanoscale, where materials exhibit unique properties that are significantly different from their larger counterparts. These properties can be exploited for a wide range of applications, including medicine. Among the emerging therapeutic approaches for the treatment of neurodegenerative diseases, nanotechnologies are gaining prominence as a promising avenue to explore. Here, we review the state of the art of biological and artificial vesicles and their biological properties in the context of neurodegenerative diseases. Indeed, nanometric structures such as extracellular vesicles and artificial vesicles represent a promising tool for the treatment of such disorders due to their size, biocompatibility, and ability to transport drugs, proteins, and genetic material across the blood–brain barrier to target specific cells and brain areas. In the future, a deeper and broader synergy between materials science, bioengineering, biology, medicine, and the discovery of new, increasingly powerful delivery systems will certainly enable a more applied use of nanotechnology in the treatment of brain disorders.

## 1. Introduction

Neurodegenerative diseases (NDs) (more than 600 pathologies), characterized by brain degeneration, lead to debilitating cognitive and motor deficits that limit normal activities. To date, NDs represent a major challenge to healthcare systems worldwide with a significant economic impact [1].

In addition to neurodegenerative diseases caused by brain injury, there are diseases caused by a variety of factors, genetic mutations, environmental factors, and metabolic problems. Several chronic neurodegenerative diseases manifest as deposits of misfolded or aggregated proteins that impair neuronal connectivity and plasticity and trigger cell death signaling pathways [2,3]. For example, the degenerating brain contains abnormal accumulations of misfolded, aggregated proteins such as α-synuclein and synphilin-1 in Parkinson’s disease (PD) and amyloid-β (Aβ) and tau in Alzheimer’s disease (AD). Other disorders in which protein aggregation is seen include Huntington’s disease (a polyQ disorder), amyotrophic lateral sclerosis (ALS), and prion diseases [2,3]. Mechanisms common to several neurodegenerative diseases may include a rare mutation in the disease-associated gene encoding the protein, inflammation, and excessive generation of reactive nitrogen species (RNS) and reactive oxygen species (ROS), which can contribute to neuronal cell injury and death [2].

SNAREs (soluble N-ethylmaleimide-sensitive factor attachment protein receptors) are a diverse family of proteins essential for synaptic vesicle exocytosis and synaptic trans-mission. These proteins are crucial for various fundamental neuronal functions, including neurite initiation and growth, axonal specification and extension, synaptogenesis, and synaptic transmission [4]. In fact, the inhibition of the SNARE complex formation, defects in SNARE-dependent exocytosis, and the impaired regulation of SNARE-mediated vesicle fusion have been linked to neurodegeneration [4].

Syntaxin-5 is a Qa SNARE that is ubiquitously expressed and plays an important function in the endoplasmic reticulum (ER), regulating membrane fusion in the ER and Golgi apparatus. Its overexpression has been linked to APP accumulation and reduced Aβ42 secretion and is associated with neurodegeneration. SNAP-25 is present in the cerebrospinal fluid (CSF) and has been linked to synaptic damage that precedes neuronal loss and impaired memory formation in AD [4]. Specifically, the SNAP-25/Aβ42 ratio was increased in patients with progressive mild cognitive impairment and AD compared to cognitively sound individuals [4].

Syntaxin-binding protein 6 (STXBP6), also known as amysin, is an essential component of the SNAP receptor (SNARE) complex. The truncated protein in the STXBP6 gene, resulting in a premature stop codon, could negatively modulate the exocytosis of synaptic vesicles. A plausible, robust correlation between the deletion of the STXBP6 gene and the manifestation of developmental epileptic encephalopathy has been observed [5].

Activation of the proteasomal degradation of misfolded proteins has been proposed as a therapy for neurodegenerative diseases, but its role is uncertain. Surprisingly, proteasome inhibitors alleviated neurodegeneration in CSPa-deficient mice, improved SNARE complex assembly, and extended their lifespan. These findings suggest that proteasome inhibition may be a therapeutic strategy for some NDs [6]. The SNARE complex is therefore a promising therapeutic target for several neurodegenerative and neurological diseases.

**Figure 1:**
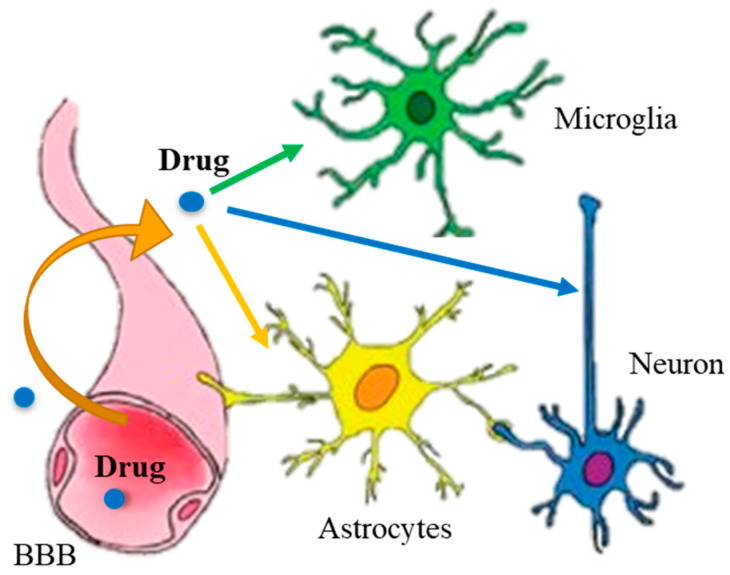
Therapeutic agents circulating in a blood stream to counteract brain pathologies not only need to cross the blood–brain barrier (BBB) but it is also very important that the formulations selectively target specific brain regions and cells (neurons, microglia, or astrocytes) involved in different brain diseases.

Previous therapeutic strategies based on pharmaceutical drugs targeting inflammation, oxidative stress, and neurodegeneration mechanisms faced challenges primarily due to difficulties in effective drug delivery. The main obstacle to the treatment of NDs is not only the intrinsic complexity of the nervous system but also the presence of biological barriers (the blood–brain barrier (BBB) and the blood–cerebrospinal fluid barrier). The BBB in the brain serves as a crucial protective shield for maintaining normal brain function and effectively protects the brain from harmful substances. The blood–brain barrier (BBB) is made up of several key components: endothelial cells, which form the walls of the cerebral capillaries and are tightly connected to each other; a basement membrane, which supports the endothelial cells and provides an additional layer of protection; astrocytes, which provide biochemical support to the endothelial cells; and pericytes, which surround the endothelial cells. Most macromolecules are unable to penetrate the cerebral endothelium; only small molecules with a molecular weight of less than 400 Da can cross the BBB. Therefore, these barriers reduce the accessibility and efficacy of therapies (BBB stops 95% of potential drugs), with a number of side effects resulting from the distribution of drugs in the body that cannot penetrate the central nervous system (CNS) [7,8]. It is also important that therapeutic agents reach the brain lesion without affecting other areas of the CNS to avoid adverse effects. Finally, it is very important that formulations selectively target specific brain cells (neurons, microglia, oligodendrocytes, or astrocytes) involved in different brain pathologies (Figure 1).

Steps towards enhancing the future of medicine are leading to the development of new therapeutic approaches, such as micro- and/or nanovesicles, which fall within the fields of nanomedicine and bioengineering [9,10].

**Figure 2:**
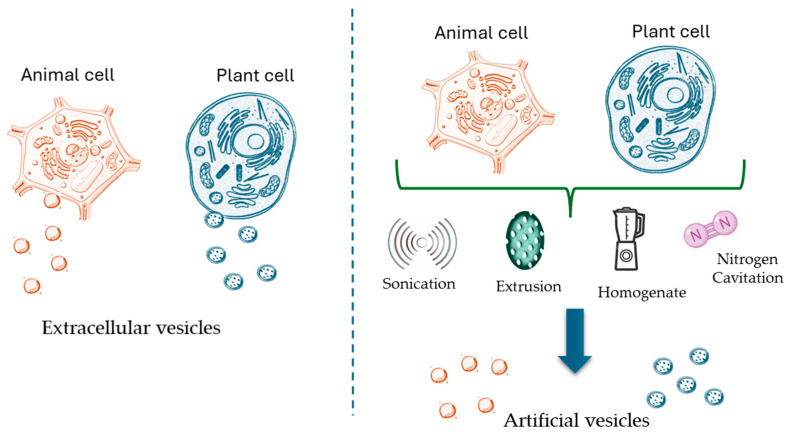
Schematic representations of the different types of vesicles (extracellular vesicles and artificial vesicles) obtained from animal and plant cells.

Extracellular vesicles (EVs) and artificial vesicles (AVs) are emerging tools in nanomedicine. In fact, due to their ability to load and deliver drugs and regulatory gene sequences, they have been extensively studied for the treatment of brain diseases, demonstrating biological effects in counteracting neurodegenerative processes in various animal models [11]. EVs are cell-derived membrane structures released by mammalian, plant, and bacterial cells that form a cellular communication system (Figure 2). It has been shown that all types of brain cells (neurons, oligodendrocytes, astrocytes, and microglia) produce extracellular vesicles. EVs can transport different types of molecules (nucleic acids and proteins), which can influence the functional biological properties of recipient cells; indeed, in the CNS, EVs can influence neurodevelopment, myelination, regeneration, and synaptic activity [12]. Thanks to their content, EVs could represent powerful biomarkers for neurodegenerative diseases [13], or, since they constitute an intercellular communication system and containing regulatory molecules, they could also be candidates for therapeutic use or could represent a brain drug delivery system [14].

AVs that mimic natural vesicles can consist of an aqueous compartment physically separated from its environment by an artificial membrane, (liposomes and polymersomes) [11], or artificial vesicles can be derived from the destruction of cells whose membranes reassemble to form nano- or microvesicular structures [10]. Specifically, the biofabrication methods currently available for the production of cell-derived AVs are mainly based on several techniques such as sonication, extrusion, homogenization, and cavitation (Figure 2).

The approach of nanobiotechnologies, in particular EVs and AVs, could offer unique opportunities to (i) deliver molecules (RNA, such as miRNA or siRNA and/or proteins) or load drugs by increasing the local drug concentration in diseased tissues while reducing accumulation in other tissues and organs (e.g., the liver or kidneys); (ii) improve drug bioavailability, stability (the protection of a drug from degradation), and solubility; (iii) overcome physiological barriers; (iv) enable targeted delivery and controlled release; (v) prolong circulation times; and (vi) reduce doses and frequency of administration by limiting potential adverse effects. All of these advantages would increase the efficacy of therapies for the treatment of brain disorders [9,15].

## 2. Extracellular Vesicles in Neurodegenerative Diseases

Extracellular vesicles (EVs), including exosomes and microvesicles, have emerged as promising tools in the treatment and diagnosis of neurodegenerative diseases due to their role in intercellular communication and their ability to traverse the BBB. In recent years, research on EVs has expanded greatly, in part due to recent innovations in analytical imaging techniques that have greatly increased our understanding of how the chemical structure and function of vesicles contribute to biological roles such as compartmentalization, storage, and molecular trafficking. EVs are naturally secreted by neurons, glial cells, and other cell types, playing a crucial role in the regulation of cellular functions. Importantly, they can carry bioactive molecules such as proteins, lipids, and RNA (miRNA or siRNA), making them suitable for the targeted delivery of therapeutic agents to the brain. Studies have demonstrated that EVs can deliver siRNA and other small molecules across the blood–brain barrier to modify disease pathways in conditions such as Alzheimer’s (AD) and Parkinson’s diseases (PD) and multiple sclerosis (MS) [15,16]. For instance, Alvarez-Erviti et al. successfully used exosomes to deliver α-synuclein-targeting siRNA in a mouse model of Parkinson’s disease, reducing α-synuclein aggregates and ameliorating disease symptoms. Additionally, EVs are involved in the clearance of pathological proteins, such as amyloid-β (Aβ), which accumulate in the brain during AD. Research by Yuyama et al. [17] has shown that neuronal exosomes may sequester and aid in the clearance of Aβ, suggesting a potential neuroprotective role for EVs in mitigating Alzheimer’s pathology. Furthermore, exosomes may not only clear toxic proteins but also propagate neurodegenerative pathology. Sardar Sinha et al. [18] demonstrated that exosomes containing toxic amyloid-β oligomers can spread the pathological hallmark of AD, thereby accelerating disease progression. A particularly promising aspect of EVs is their role in promoting remyelination, the process of restoring myelin sheaths damaged by neurodegenerative diseases such as MS. Several studies have highlighted the potential of EVs, particularly those derived from mesenchymal stem cells (MSCs) and oligodendrocytes, to facilitate myelin regeneration. EVs have been shown to carry key molecules such as microRNAs and growth factors that enhance oligodendrocyte precursor cell (OPC) maturation and differentiation, leading to the restoration of myelin. A study by Pusic and Kraig [19] demonstrated that exosomes derived from MSCs contain specific miRNAs that promote remyelination in mouse models of demyelination, thereby indicating a novel therapeutic strategy for diseases like MS. Additionally, research by Lopez-Verrilli and Court [20] has shown that EVs derived from Schwann cells can support axonal regeneration and potentially enhance remyelination, providing further evidence of the regenerative potential of EVs in the nervous system. Beyond their role in disease modulation, EVs also hold potential as delivery vehicles for therapeutic interventions. The capacity of EVs to encapsulate and deliver anti-inflammatory drugs directly to the brain, offering a novel route for treating neuroinflammatory diseases. Given that neurodegenerative diseases, such as MS, AD, and amyotrophic lateral sclerosis (ALS), are often accompanied by inflammation, the anti-inflammatory properties of EVs could be harnessed to alleviate symptoms and slow disease progression. Moreover, the ability of EVs to facilitate communication between cells in the central nervous system (CNS) presents further opportunities for therapeutic interventions. Rajendran and Bali [21] emphasized how EVs mediate the transfer of proteins, lipids, and nucleic acids between neurons and glial cells, underscoring their role in the progression of neurodegenerative diseases. Similarly, Budnik et al. [22] underscored the importance of EVs in maintaining homeostasis in the CNS, suggesting that their dysregulation could contribute to neurodegenerative disorders. Lombardi and coworkers showed that proinflammatory microglia release EVs that block the remyelination process, whereas microglia in co-culture with immunosuppressive mesenchymal stem cells release EVs that induce myelin repair by promoting the recruitment of oligodendrocyte progenitor cells (OPCs) [23]. Furthermore, EVs derived from IFN-stimulated dendritic cells have been shown to contain microRNAs that promote remyelination after lysolecithin injury. In particular, EVs stimulated OPCs in myelin-forming cells and reduced oxidative stress [24]. BV-2 microglial cells were engineered to release EVs containing lactadherin (Mfg-e8) and cytokine IL-4 as an anti-inflammatory agent. These EVs modulated inflammation by significantly reducing the clinical signs of the experimental autoimmune encephalomyelitis (EAE) model of MS [25]. Curcumin-loaded exosomes (Exo-Cur) administered intranasally enabled the rapid delivery of the encapsulated drug to the brain, preventing lipopolysaccharide (LPS)-induced inflammation and reducing the severity of the MOG-induced EAE model [26,27].

A study by Upadhya R. [28] revealed the presence of several key proteins (hemopexin, agrin, PTX3, Gal-3BP, and nidogen-1) recognized for their neuroprotective effect in extracellular vesicles (EVs) released by neural stem cells (hiPSC-NSCs). In particular, hemopexin (Hpx), which is expressed in all regions of the CNS, plays a role in maintaining the integrity of the BBB, promoting angiogenesis, synaptic plasticity, and synaptogenesis [29,30]. Furthermore, it inhibits neuroinflammation by regulating the polarization of proinflammatory M1 microglia into anti-inflammatory M2 microglia via the low-density lipoprotein receptor-related protein 1 (LRP-1) [31]. EVs derived from hiPSC-NSCs have been observed to exert a rapid and pronounced effect on neurons, microglia, and a subset of astrocytes across all regions of the brain in an animal model of chronic neuroinflammation. Moreover, the same study identified the presence of eight miRNAs in hiPSC-NSC-EVs (miR-320a, 320b, 103a-3p, 21-5p, 26a-5p, 30a-3p, 181a-5p, 191-5p) that have a protective effect against various pathogenetic mechanisms in AD and PD models [28].

Mesenchymal stem-cell-derived extracellular vesicles (MSC-EVs) have been applied in several studies involving neurodegenerative disorders due to their ability to cross the BBB by exerting therapeutic effects in the CNS mediated by their content [32]. In this regard, Sha S. [33] showed that bone marrow MSC-EVs (BM-MSC-EVs) transport microRNAs such as miR-29c-3p in hippocampal neurons in a mouse model of AD. It has been shown that deregulation of the Wnt/β-catenin pathway and elevated levels of Aβ1-42 are involved in the onset and progression of AD. miR-29c-3p is able to downregulate the BACE1 (beta-secretase 1) gene by activating the Wnt/β-catenin pathway, thereby reducing the levels of Aβ1-42 and certain inflammatory cytokines (IL-1β, IL-6, and TNF-α), playing a therapeutic role in the treatment of AD [33].

The growing body of evidence on EVs highlights their dual potential as both biomarkers and therapeutic tools for neurodegenerative diseases. As outlined by Ravichandran and Prasad [27], the ability to detect specific EV signatures in bodily fluids, such as cerebrospinal fluid or blood, provides an exciting avenue for the early diagnosis and monitoring of diseases like Alzheimer’s and amyotrophic lateral sclerosis (ALS). This capability, combined with their ability to deliver therapeutics across the BBB, positions EVs as powerful assets in personalized medicine approaches for treating neurodegenerative conditions. However, for biomedical applications of EVs, there is an urgent need to establish their safety, source (patient cell culture), isolation protocols, reproducibility, and characterization. Indeed, the composition of EVs depends on the physiological state of the cells. Therefore, new approaches for the efficient and reproducible production of EVs will be needed in the coming years. To date, yield and heterogeneity issues have hindered the clinical use of EVs [7].

Each stage of the process, from isolation to administration, presents a range of challenges and limitations. The high structural complexity of these vesicles, coupled with their extreme heterogeneity, renders the establishment of a single isolation protocol that is equally adequate and productive an impossibility. The isolation methods described in the literature include differential and density gradient centrifugation, filtration, size-exclusion chromatography, and precipitation. Differential centrifugation, which employs a succession of centrifugations to remove debris and dead cells, is one of the most popular techniques [34]. Nevertheless, despite the advantages of this methodology in terms of speed, cost-effectiveness, and yield compared to density gradient centrifugation, it is important to note that it also has the disadvantage of requiring highly expensive machinery and achieving lower purity [35]. One technique used to obtain a purer product is size exclusion chromatography (SEC), which separates particles according to their size [36]. Another widely used alternative involves the use of membranes which, based on different pore sizes, allow particles with specific sizes or molecular weights to be selected [37]. The choice of one specific separation method over another will therefore depend on the known properties of the specific EV sources and the desired EV yield and specificity. The subsequent phase of this study entails the characterization of EVs, with the objective of acquiring data regarding their quantity, size, component composition, and biochemical properties. The combination of electron microscopy techniques and mass spectrometry enables the structure, morphology, and composition of vesicles to be studied [38,39]. Once more, nanoparticle tracking analysis (NTA) or flow cytometry evaluate the size and concentration of vesicles [40,41]. Unfortunately, the small and heterogeneous particle size, the lack of universal identification methods, and the lack of specificity of the techniques used act as obstacles to adequate characterization. It is evident that no single technique is capable of meeting the comprehensive requirements for a complete characterization of vesicles. Conversely, the utilization of measurements employing orthogonal methodologies for the same parameter could circumvent the inherent biases intrinsic to the techniques themselves. Once the vesicles have been properly characterized, they can be conjugated with therapeutic agents through the use of techniques such as electroporation. This involves the application of electrical impulses to temporarily permeate the vesicle membrane, thereby allowing the drug to enter and be absorbed [42]. However, this method carries the risk of damaging the vesicle membrane and reducing its stability. An alternative approach could be passive incubation of the drug, although this is often associated with low loading efficiency. The final stage in the development of EVs-based therapies is the administration of the vesicles. This can be achieved via three main routes: intravenous, intranasal, or intrathecal [43]. To date, intranasal administration has demonstrated considerable promise, offering a direct route to the central nervous system via the olfactory nerve, thereby circumventing the BBB.

The potential of EVs as a therapeutic agent has been acknowledged by the International Society for Extracellular Vesicles (ISEV), which has collated comprehensive manuscripts entitled Minimal Information for Studies of Extracellular Vesicles. These manuscripts outline the advantages, limitations, production methods, separation techniques, and characterization of EVs in a clear, comprehensive, and up-to-date manner [44,45].

## 3. Plant-Derived Extracellular Vesicles

Plant-derived extracellular vesicles (PEVs) are nanoscale vesicles that can be isolated from various plant tissues, including fruits, roots, seeds, and leaves, using standardized isolation and purification techniques. Of these methods, ultracentrifugation is the most commonly used, although other approaches such as differential centrifugation, sucrose density gradient centrifugation, and filtration are also widely used [46]. Fruits, vegetables, and spices have emerged as prime sources for the production of plant-based EVs. For instance, species such as grapes, grapefruit (*Citrus* × *paradisi*) [47,48], ginger (*Zingiber officinale*) [49], orange (*Citrus sinensis*), lemon (*Citrus* × *limon*) [50], tomato (*Solanum lycopersicum*) [51], prickly pear (*Opuntia ficus-indica*) [52] coconut, sunflower seeds, and cactus [53] have shown great potential for PEVs’ isolation, with promising applications in drug delivery and therapeutic development. The bioactive molecules carried by these vesicles, such as proteins, lipids, and RNA (miRNA or siRNA), allow them to modulate cellular functions and provide health benefits, including anti-inflammatory, anti-cancer, and regenerative effects [54].

PEVs are rich in microRNAs (miRNAs), a group of small non-coding RNAs that play critical roles in the regulation of physiological and pathological processes, including cell proliferation, apoptosis, metabolism, and immune responses. Teng et al. analyzed PEVs isolated from carrot, garlic, grape, and ginger, focusing on the presence of RNA. They found that these PEVs contain small RNAs, including miRNAs, that can influence the composition of the microbiome and suppress inflammatory conditions such as colitis. This suggests that EVs of plant origin ingested with food may play a role in modulating the gut microbiota [55]. Several studies have further investigated the effects of specific plant miRNAs, such as mi168a and miR159, found in edible plants and demonstrated their ability to regulate gene expression in mammals after ingestion. In addition, a comprehensive investigation by Xiao et al. examined the miRNA profiles of eleven different fruits and vegetables and identified several miRNAs that were shown to influence the expression of genes related to cancer and inflammatory cytokines in vitro. These findings highlight the potential of PEV miRNAs to influence human health through dietary intake [51].

PEVs have shown significant therapeutic powers in relation to their biological cargo [56] and, as a matter of fact, it is possible to use their nucleic acids, bioactive lipids, or cell surface proteins, or they can be applied as delivery systems for other active ingredients, due to their biocompatibility and adjustable nature [46]. As a consequence, plenty of PEV applications have been studied and, among them, plant-derived extracellular vesicles may be very efficient nutritional carriers if fruits and/or vegetables are included in someone’s diet and used as oral drug delivery systems. Furthermore, PEVs have been proved in oral administration for the treatment of various diseases, such as colitis [48], bowel disease [57], liver disease [58], brain tumor progression [59], and encephalitis [60]; in this regard, it is relevant to underline that this pathogenesis was strongly related to the dysfunction of the gut barrier and the dysbiosis of the gut microbiota. Furthermore, the gut–brain axis is necessary for the transduction of detrimental signals from the gut to the brain, and for instance, inflammatory factors resulting from leaky gut penetrate the BBB, causing the consequent destruction of its integrity. As a result, the activated gut immune cells may be translocated to the brain to amplify neuroinflammation and inflammatory factors transmitted through the vagal nerve which connects the gut and the brain, resulting in increased neuroinflammation. It is so evident that a tight relation exists between the gut and the brain, so any kinds of PEVs that are effective for inflammatory gut diseases might also be useful for neurodegenerative diseases targeted by neuroinflammation hallmarks. The possible mechanism of a therapeutic role of PEVs in the treatment of neurodegenerative diseases is obtained thanks to the balance of gut microorganisms’ composition, preventing, in this way, the peripheral inflammatory factors entering the brain and diminishing neuroinflammation. In fact, it is widely accepted that microglia play a pivotal role in the progression of Alzheimer’s disease (AD) [61]. In particular, physiological proliferation, chemotaxis, and phagocytosis processes are usually required to remove excessive Aβ protein deposition; on the other hand, over-activated microglia would release inflammatory cytokines to induce neuronal death [62].

Oxidative stress can also damage the BBB, contributing to neurological diseases such as Parkinson’s disease, ischemic stroke, and inflammatory brain disorders. Oxidative stress can be induced by ROS proteins, lipids, and DNA, ultimately compromising the integrity of the BBB. Recent evidence has shown that PEVs, such as those from lemon, strawberry, and carrot, offer protection against oxidative stress [63,64,65].

Drug delivery across the BBB is challenging due to the tight structure of the barrier and limited tumor penetration. Nanoparticle-based drug delivery systems have been developed to address this issue, offering advantages such as high drug loading, functionalization, and controlled release. However, synthetic nanoparticles often struggle with prolonged circulation and effective BBB penetration. In contrast, PEVs possess natural membrane proteins that facilitate cellular uptake and crossing physiological barriers such as the BBB. Novel approaches using PEVs have shown promise in bypassing the BBB [66]. For example, grapefruit-derived EVs have been used to deliver drugs to glioma tissue via receptor-mediated transport and membrane fusion, enhancing cellular internalization and antiproliferative effects. Additionally, researchers have explored non-invasive methods such as intranasal delivery using folic acid (FA)-coated grapefruit-derived nanocarriers to deliver therapeutic miRNAs across the BBB. This method improves drug absorption and reduces toxicity, offering potential for the treatment of brain diseases [59,67].

One study showed that EVs obtained from Drynariae Rhizoma roots were enriched in the enzyme NAD(P)H-quinone oxidoreductase, widely distributed in eukaryotic cells and recognized for its protective mechanism against quinone-induced oxidative damage in the oxidative phosphorylation pathway [68], suggesting a protective role in oxidative stress-associated disorders such as AD, PD, and in Huntington’s disease (HD) [69]. Furthermore, Vestuto V. [70] highlighted the presence in EVs of Salvia sclarea and Salvia dominica of eight proteins homologous to their human counterparts with >50% sequence identity. These include GRP75 (a 75 kDA glucose-regulated protein), which plays a key role in mitochondrial homeostasis [71]. Furthermore, plants naturally carry EVs containing small RNAs that play a key role in plants’ natural defense system against infection [72]. Interesting work has described certain miRNAs present in EVs isolated from apple, in particular miR-125, miR-146a, and miR-146b, which are recognized for their anti-inflammatory effect both in vivo and in vitro [73].

## 4. Artificial Vesicles

As our understanding of the biological properties of extracellular vesicles and their physiological functions deepens, increasingly elegant artificial vesicles with excellent physiological properties and functions are being developed for a wide range of applications. In particular, artificial vesicles (AVs) have emerged as an innovation in the fields of biotechnology and nanomedicine. These synthetic vesicles are inspired by natural extracellular vesicles, such as exosomes and microvesicles. AVs offer the possibility of encapsulating bioactive molecules, such as proteins, nucleic acids, or drugs, for targeted delivery to specific tissues. These synthetic or bioengineered vesicles are designed to mimic the functional and structural properties of natural vesicles, with the added advantage of greater control over size, composition, and surface modifications [74]. This unique flexibility makes AVs a powerful tool to address otherwise difficult-to-treat diseases, particularly neurodegenerative diseases, for which targeted delivery to the central nervous system is crucial.

AVs are obtained through a variety of techniques that allow for precise control over their size, composition, and functional properties. The three primary methods for generating AVs are bottom-up synthesis, top-down synthesis, and hybrid vesicle formation. In the bottom-up synthesis approach, synthetic vesicles are constructed from basic molecular components, such as lipids, polymers, and proteins. The most common method involves the self-assembly of phospholipids into liposomes, which resemble natural cell membranes (Figure 2). By manipulating the lipid composition of these vesicles, properties such as their size, surface charge, and encapsulation efficiency can be modified [75,76,77]. In contrast, the top-down methods start with whole cells, which are mechanically or chemically disrupted to form vesicles. For example, plasma membranes can be extracted or sonicated to form membrane-derived vesicles that retain functional surface proteins and receptors. These vesicles can mimic the behavior of natural extracellular vesicles and can be functionalized to carry therapeutic agents [78]. The hybrid vesicle formation method combines the best features of natural and synthetic systems. Hybrid vesicles are formed by fusing synthetic liposomes or polymersomes with natural extracellular vesicles. These vesicles can possess the targeting capabilities of natural EVs while being engineered for stability and cargo capacity. Hybrid vesicles allow for modifying the surface with targeting ligands or therapeutic molecules, enhancing their application in neurodegenerative disease treatment [79].

AVs offer potential solutions by facilitating targeted drug delivery through the BBB, improving bioavailability and reducing off-target effects [80,81]. Indeed, after formation, AVs are functionalized with targeting ligands, such as peptides or antibodies, to facilitate their uptake by specific cell types. These ligands can be designed to target receptors on the surface of neuronal cells or endothelial cells of the BBB, improving the specificity and efficiency of delivery. All of these aspects have made artificial vesicles a promising resource for the treatment of neurodegenerative brain diseases as they can bypass the BBB and deliver therapeutic agents directly to the affected neurons.

**Figure 3:**
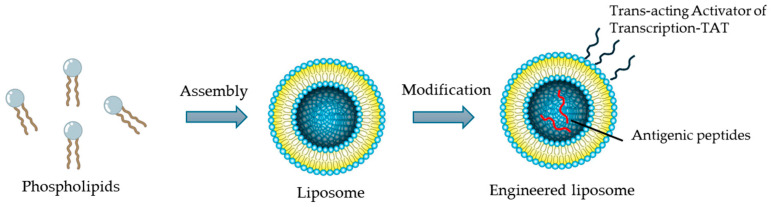
Schematic of liposome assembly and encapsulation of therapeutic molecules.

Liposomes were the first drug delivery system to be investigated and are approved by the Food and Drug Administration (FDA) as a therapeutic drug delivery system [82]. Such systems protect loaded molecules from degradation and allow for specific targeting [83]. Indeed, liposomes have been modified with cell-penetrating peptides (trans-acting activator of transcription—TAT) to cross the BBB and increase drug concentration in the CNS [84] (Figure 3). In addition, several data have demonstrated the therapeutic potential of liposomes encapsulated with myelin antigenic peptides in MS to induce immune tolerance (Figure 2).

Alzheimer’s disease is characterized by the accumulation of amyloid-beta plaques and tau tangles, which disrupt synaptic function and lead to cognitive decline. AVs can be loaded with small interfering RNA (siRNA)-targeting beta-secretase (BACE1), an enzyme involved in amyloid-beta production, to reduce plaque formation. Studies have shown that AVs can successfully deliver siRNA across the BBB and into neurons, reducing amyloid-beta levels and slowing down disease progression in animal models [85,86].

Moreover, oxidative stress and mitochondrial dysfunction are among the early events of AD, which trigger neurodegeneration. The use of natural antioxidants constitutes a neuroprotective strategy. To this end, solid lipid nanoparticles containing ferulic acid (FA-SNL), a natural molecule with important antioxidant properties, have been developed. Treatment with FA-SNL reduced ROS generation and restored neuronal mitochondrial functionality [87].

In addition to gene silencing, AVs can encapsulate anti-inflammatory drugs or neurotrophic factors like BDNF (brain-derived neurotrophic factor), providing neuroprotection and promoting neuronal survival [88]. Furthermore, in Parkinson’s disease, AVs loaded with siRNA- or miRNA-targeting alpha-synuclein expression have been shown to reduce its aggregation and protect dopaminergic neurons from degeneration [89]. This targeted approach could slow down the progression of PD and mitigate motor symptoms in patients.

Nanovesicles derived from neutrophil membranes were used to deliver Resolvin D2 (a molecule derived from docosahexaenoic acid) to mice with ischemic stroke. In particular, using nitrogen cavitation and inspired by the binding of neutrophils to the endothelium during stroke, the authors generated nanovesicles derived from HL-60 cells differentiated into neutrophils. By interacting with the inflamed brain endothelium, the nanovesicles attenuated neuroinflammation [90].

Nanovesicles derived from macrophage membranes loaded with nerve growth factor were generated by extrusion through the polycarbonate membrane [91]. These nanovesicles efficiently delivered nerve growth factor into the spinal cord lesion, exerting a neuroprotective effect [91].

We have recently bio-fabricated nanovesicles from brain myelin as a new potential brain-to-brain delivery system [92]. In particular, myelin nanovesicles produced with a simple, efficient, inexpensive, and reproducible production protocol exhibit high stability and cytocompatibility and are able to load drugs and reach the CNS after nasal administration [93]. They are also able to interact preferentially with microglial cells by modulating the inflammatory process [93].

Nanovesicles derived from brain endothelial cells by serial extrusion are a good nanocarrier alternative to exosomes [94]. In particular, loaded doxorubicin has been shown to cross the BBB with an ability to target glioblastoma and have anti-tumor effects. The yield in nanovesicle production is much higher (500-fold) than with exosomes.

Liposomes rich in phosphatidylserine (PS) (PS–liposomes), a component present in apoptotic cells that modulates immune responses, were also prepared. PS–liposomes, which are recognized by dendritic cell receptors, were loaded with the peptide MOG40-55 (an MS autoantigen) and tested in a mouse model of MS, where they induced immune tolerance [95].

Synaptosomes, a subcellular fraction isolated from synaptic terminals, have been pro-posed as vesicles for the delivery of mitochondria (mitochondrial transfer) into neuronal cells [96]. Synaptosomes could be used for the treatment of many brain diseases characterized by mitochondrial dysfunction, such as Alzheimer’s disease, Parkinson’s disease, etc. [96].

In addition to artificial vesicles derived from synthetic materials or mammalian cells, artificial plant-derived vesicles (APDVs) are emerging as a novel, natural alternative for therapeutic delivery. These vesicles, isolated from edible plants such as ginger, grapes, and grapefruit following the destruction of plant tissue, have properties similar to those of mammalian extracellular vesicles and can be used to treat neurodegenerative diseases [97]. Like AVs, APDVs have the potential to cross the BBB. Studies have shown that vesicles derived from grapefruit and ginger can traverse the BBB in animal models, making them suitable candidates for delivering therapeutics to the brain [98]. Moreover, APDVs can be functionalized with targeting molecules to enhance their delivery efficiency to neuronal cells. Furthermore, APDVs can be loaded with natural compounds that have anti-inflammatory and neuroprotective effects. For instance, ginger-derived vesicles loaded with curcumin have been shown to protect neurons from oxidative stress and inflammation, which are key contributors to neurodegeneration in diseases like AD and PD [99]. Similarly, grape-derived vesicles can be used to deliver miRNA that modulates the gene expression involved in neuroinflammation and protein aggregation, providing a novel approach to treating these conditions [59]. APDVs from bitter gourd, specifically miRNA5266, were found to reduce BBB damage by inhibiting the expression of matrix metalloproteinase-9 (MMP-9), thereby preventing ischemia–reperfusion injury and neuronal apoptosis [100]. 

The therapeutic mechanisms of EVs, PEVs and AVs in neurodegenerative diseases are summarized in Table 1.

## 5. Conclusions

To conclude, extracellular vesicles and artificial vesicles hold great promise as therapeutic agents in the context of neurodegenerative diseases.

Their ability to cross the BBB and carry diverse molecular cargoes positions them as versatile tools in the treatment of neurodegenerative diseases such as Alzheimer’s, Parkinson’s, ALS, and multiple sclerosis. Extracellular vesicles are naturally occurring and have advantages such as biocompatibility and the ability to normally cross biological barriers, but for biomedical applications, there is an urgent need to establish their safety, source (patient cell culture), isolation protocols, reproducibility, and characterization.

Thus, due to the inherently complex biogenesis of EVs and their wide heterogeneity in size, composition, and origin, the study of EVs has remained challenging to date. Artificial vesicles, on the other hand, are man-made and can be engineered with the advantage of greater control over size, composition, and surface modifications, but the safety profile needs to be addressed. However, for both EVs and AVs, further studies are needed to fully understand their mechanisms of action, to optimize their isolation and/or production methods, and to ensure their efficacy in clinical applications.

## Figures and Tables

**Table 1:** Therapeutic mechanisms of extracellular vesicles (evs), plant-derived extracellular vesicles (pevs), and artificial vesicles (AVs) in neurodegenerative diseases. This table summarizes the therapeutic actions of various vesicle types—EVs, PEVs, and AVs—in Alzheimer’s disease (AD), Parkinson’s disease (PD), and multiple sclerosis (MS).

Vesicle Type	Disease	Therapeutic Mechanism	Detailed Mechanisms/Effects	Ref.
Extracellular vesicles (EVs)	AD	Sequestration and clearance of Aβ	Neuronal EVs can sequester amyloid-β (Aβ), aiding its clearance and potentially slowing down AD progression	[17]
		Propagation of neurodegenerative pathology	Exosomes containing toxic Aβ oligomers may also propagate AD pathology by spreading these oligomers	[18]
		Neuroprotection via anti-Inflammatory delivery	EVs carrying anti-inflammatory agents like curcumin reduce neuroinflammation in AD models	[26]
	PD	Delivery of α-synuclein-targeting siRNA	EVs can cross the BBB to deliver siRNA targeting α-synuclein, reducing toxic protein aggregates	[16]
		Propagation of toxic protein aggregates	EVs may inadvertently spread misfolded α-synuclein across neurons, accelerating disease progression	[21]
	MS	Promotion of remyelination	EVs derived from MSCs contain miRNAs and growth factors that stimulate OPC maturation and myelination	[19,20]
		Immune modulation	EVs carrying IL-4 reduce neuroinflammation in MS, alleviating symptoms in models of MS	[25]
		Antioxidative effects	EVs modulate oxidative stress, protecting the blood–brain barrier (BBB) and reducing CNS damage	[26]
Plant-derived extracellular vesicles (PEVs)	AD	Oxidative stress reduction	PEVs from lemon, strawberry, and carrot reduce oxidative stress, protecting neurons	[63,64,65]
		Gut–brain axis modulation	PEVs improve gut health, preventing peripheral inflammation from affecting the brain and reducing neuroinflammation in AD	[61]
	PD	Anti-inflammatory and neuroprotective effects	Ginger-derived PEVs loaded with curcumin reduce neuroinflammation, slowing down PD progression	[99]
		Modulation of neuroinflammation via miRNAs	Grape and ginger PEVs deliver miRNAs to modulate gene expression involved in inflammation and protect dopaminergic neurons	[89]
	MS	Modulation of gut microbiota for reduced CNS inflammation	By modulating the gut microbiome, PEVs reduce inflammatory factors that contribute to MS progression	[55]
		Antioxidant protection of BBB	PEVs protect BBB integrity through antioxidative effects, potentially preventing neuroinflammation	[67]
Artificial vesicles (AVs)	AD	siRNA delivery targeting BACE1	AVs are loaded with BACE1-targeting siRNA to reduce amyloid-beta plaque formation in the brain	[94]
		Delivery of antioxidant agents	Solid lipid nanoparticles containing ferulic acid combat oxidative stress, preserving neuronal health	[87]
		Encapsulation of neurotrophic factors	AVs can deliver BDNF to enhance neuron survival and prevent synaptic loss, alleviating AD symptoms	[88]
	PD	siRNA/miRNA delivery targeting α-Synuclein	AVs reduce α-synuclein aggregation, protecting neurons from degeneration associated with PD	[89]
		Delivery of anti-inflammatory agents	Encapsulation of neurotrophic and anti-inflammatory molecules mitigates neuroinflammation in PD	[90]
	MS	Delivery of myelin antigenic peptides for immune tolerance	AVs encapsulating myelin peptides promote immune tolerance, reducing autoimmune response in MS	[93]
		Targeted delivery of anti-inflammatory agents	AVs functionalized with immune-modulatory agents specifically target and reduce inflammation in MS	[95]
